# Creating the blueprint for a national UK undergraduate plastic surgery curriculum

**DOI:** 10.1016/j.jpra.2026.09.023

**Published:** 2026-09-23

**Authors:** Shubham Gupta, Richard Clough, Anna Rose, Kevin Zarabian, Ruby Cummings, Berivan Macit, Alexander James Baldwin, Aidan Fitzgerald, Paul McArthur

**Affiliations:** a PLASTICS UK Research Collaborative, London, United Kingdom; b Plastic Surgery Trainees Association (PLASTA UK), United Kingdom; c The Royal Marsden Hospital NHS Foundation Trust, London, United Kingdom; d Canniesburn Regional Plastic Surgery Unit, Glasgow, G4 0SF, United Kingdom; e Plastic Reconstructive and Aesthetic Surgery Students Association, United Kingdom; f The Manchester Centre for Plastic Surgery and Burns, Manchester University NHS Foundation Trust, Manchester, United Kingdom; g Blond McIndoe Laboratories, Faculty of Biology, Medicine and Health, University of Manchester, Manchester, United Kingdom; h Department of Plastic Surgery, Royal Hallamshire Hospital, Sheffield, United Kingdom; i British Association of Plastic Reconstructive and Aesthetic Surgeons (BAPRAS) Council, London, United Kingdom

**Keywords:** Medical education, Plastic surgery, Postgraduate, Training, Undergraduate

Plastic surgery encompasses numerous conditions that overlap with many disciplines, including sarcoma, skin cancer, trauma, wound care and necrotising fasciitis. This versatility means the specialty can offer a complementary role in preparing future general practice and emergency medicine trainees. Moreover, with its broad anatomical coverage, tissue handling emphasis and application of an armamentarium of surgical techniques rather than specific procedural steps, the specialty can provide a robust foundational generalist training opportunity for those wishing to pursue any surgical speciality.

However, recent national data shows that United Kingdom (UK) undergraduate plastic surgery education is inconsistent, with most students lacking formal teaching and only 6.2% reporting a clinical rotation.^1^, ^2^ Most medical students are receptive to the idea of having greater curricular exposure (68.4%) regardless of their career intentions.^1^ The UK Medical Licensing Assessment (UKMLA) content map has standardised national undergraduate medical education, but surgery is represented in just 1 of 24 areas of clinical practice, and only 1 of 29 listed surgical conditions directly pertains to plastic surgery (breast cancer).^3^ Additionally, it simply lists topics, without elaborating on learning objectives, depth or minimum competency and some conditions whose management usually occurs under the umbrella of plastic surgery such as burns, necrotising fasciitis and peripheral nerve injury are attributed to the care of other specialties. Despite its broad clinical scope, plastic surgery is not identified as the primary specialty for any of the 36 surgical conditions listed in the Royal College of Surgeons National Undergraduate Curriculum in Surgery.^4^ However it is noted to be ‘also seen in’ 5 conditions: leg ulceration, painful/paralysed limb, breast lumps/nipple discharge, fractures and peripheral nerve injuries/palsies. Wound healing and trauma are also mentioned but not mapped to plastic surgery specifically. Given the ongoing burden of conflict-related trauma, often involving blast and thermal injuries, newly qualified doctors should possess a basic understanding of plastic surgery principles. With no clear definition of plastic surgery training at the national undergraduate level, there remains a need for a formal curriculum that sets out the minimum competencies expected of a doctor at the point of qualification.

The Plastic Surgery Trainees Association (PLASTA UK), in partnership with the British Association of Plastic Reconstructive and Aesthetic Surgeons (BAPRAS), is designing a national plastic surgery undergraduate curriculum that ensures that core tenets of plastic surgery are appropriately prioritised. The aim of the project is to perform a Delphi study by engaging with key stakeholders including plastic surgeons, general practitioners, emergency physicians and medical school deans, to produce an evidence-based consensus statement on essential areas of practice during undergraduate training. Previous work by plastic surgeons in Scandinavia has shown how this can be done successfully.^5^ Stakeholder recruitment will occur via professional networks and specialist societies with a target panel size of between thirty to sixty participants, recognising the expected attrition rates of Delphi rounds. Curriculum items will be categorised as either: ‘essential’, ‘desirable’ or ‘specialist’, with the aim of increasing inter-school uniformity and reducing variability in exposure. This undergraduate curriculum could be integrated into existing frameworks such as the National Undergraduate Curriculum in Surgery and the UKMLA content map, enabling optimal cross-linkage and consolidation with overlapping domains such as dermatology, oncology, emergency medicine and clinical skills. This may engender greater confidence in future medical graduates in dealing with basic trauma, minor injuries and burns and has the potential to have a positive patient safety impact. Other potential benefits include improvements in wound care, streamlining of referrals, and faster recognition of plastic surgery emergencies. It may also provide specialist insight as to practical mechanisms of incorporating plastic surgery within existing curricula structures such as which year groups and teaching formats might be suggested. Establishing a UK undergraduate plastic surgery curriculum would provide a consistent standard of minimum competency for graduates whilst capitalising on the diverse role of plastic surgery in medicine.

## Author contributions

Shubham Gupta: conceptualisation, literature review, manuscript drafting and revision

Richard Clough: conceptualisation, supervision

Anna Rose: supervision

Kevin Zarabian: manuscript review

Ruby Cummings: manuscript review

Berivan Macit: manuscript review

Alexander James Baldwin: supervision

Aidan Fitzgerald: supervision

Paul McArthur: supervision

## Funding

None.

## Ethical approval

n/a.

## Declaration of competing interest

None.
